# High expression of MKK3 is associated with worse clinical outcomes in African American breast cancer patients

**DOI:** 10.1186/s12967-020-02502-w

**Published:** 2020-09-01

**Authors:** Xuan Yang, Mohamed Amgad, Lee A. D. Cooper, Yuhong Du, Haian Fu, Andrey A. Ivanov

**Affiliations:** 1 Department of Pharmacology and Chemical Biology, Emory University School of Medicine, Emory University, 1510 Clifton Road, Atlanta, GA 30322 USA; 2 Emory Chemical Biology Discovery Center, Emory University School of Medicine, Emory University, Atlanta, GA USA; 3 Department of Biomedical Informatics, Emory University School of Medicine, Emory University, Atlanta, GA USA; 4 Department of Pathology, Northwestern University Feinberg School of Medicine, Chicago, IL USA; 5 Winship Cancer Institute, Emory University, Atlanta, GA USA; 6 Department of Hematology & Medical Oncology, Emory University, Atlanta, GA USA

**Keywords:** Triple-negative breast cancer, Racial disparity, Differential expression, MKK3

## Abstract

**Background:**

African American women experience a twofold higher incidence of triple-negative breast cancer (TNBC) and are 40% more likely to die from breast cancer than women of other ethnicities. However, the molecular bases for the survival disparity in breast cancer remain unclear, and no race-specific therapeutic targets have been proposed. To address this knowledge gap, we performed a systematic analysis of the relationship between gene mRNA expression and clinical outcomes determined for The Cancer Genome Atlas (TCGA) breast cancer patient cohort.

**Methods:**

The systematic differential analysis of mRNA expression integrated with the analysis of clinical outcomes was performed for 1055 samples from the breast invasive carcinoma TCGA PanCancer cohorts. A deep learning fully-convolutional model was used to determine the association between gene expression and tumor features based on breast cancer patient histopathological images.

**Results:**

We found that more than 30% of all protein-coding genes are differentially expressed in White and African American breast cancer patients. We have determined a set of 32 genes whose overexpression in African American patients strongly correlates with decreased survival of African American but not White breast cancer patients. Among those genes, the overexpression of mitogen-activated protein kinase kinase 3 (MKK3) has one of the most dramatic and race-specific negative impacts on the survival of African American patients, specifically with triple-negative breast cancer. We found that MKK3 can promote the TNBC tumorigenesis in African American patients in part by activating of the epithelial-to-mesenchymal transition induced by master regulator MYC.

**Conclusions:**

The poor clinical outcomes in African American women with breast cancer can be associated with the abnormal elevation of individual gene expression. Such genes, including those identified and prioritized in this study, could represent new targets for therapeutic intervention. A strong correlation between MKK3 overexpression, activation of its binding partner and major oncogene MYC, and worsened clinical outcomes suggests the MKK3-MYC protein–protein interaction as a new promising target to reduce racial disparity in breast cancer survival.

## Background

Breast cancer is the most common cancer and the leading cause of cancer-related death in women [1]. Recent studies have shown up to a twofold higher incidence of triple-negative breast cancer (TNBC) among African American women as compared to White women [2–4]. Moreover, African Americans die from breast cancer at up to 40% higher rate than White and Hispanic women [5–7]. The American College of Radiology (ACR) has assigned a special status for African American women at higher-than-average risk for breast cancer [8].

Previous studies have revealed significant differences in the mutation rates of several cancer driver genes in African American and White breast cancer patients (Table 1) [9–13].

**Table 1:** Frequency of tumor driver gene alterations in Black/African American and White breast cancer patients

	Black or African American	White	Oncogenic function	Regulated pathways	References
Mutation, %					
TP53	43	28	TSG	Apoptosis, senescence, DNA repair	[11]
BRCA1	10.2	6.9	TSG	DNA repair Checkpoint control	[9]
BRCA2	5.7	5.2	TSG	DNA repair, checkpoint control	[9]
PIK3CA	20	34	OG	Cell survival, proliferation	[11]
FBXW7	4.2	1.2	TSG	Cell cycle, apoptosis, differentiation	[12]
CDH1	6.4	16.2	TSG	Proliferation, adhesion, polarity, EMT	[13]
Deletion, %					
CSMD1	14.5	8.7	TSG	Proliferation, migration and invasion	[12]
RB1	8.6	4.1	TSG	Cell cycle, apoptosis	[12]
Amplification, %					
MYC	30.9	20.4	OG	Cell growth, survival, immune response, other	[12]
CCNE1	9.2	3.6	OG	Cell cycle	[12]

*TSG* tumor suppressor gene, *OG* oncogene, *EMT* epithelial-to-mesenchymal transition

For example, it was shown that African American women with at least 50% African ancestry have a higher rate of mutations in the major tumor suppressor gene TP53 (43%) as compared to White women with at least 90% European ancestry (28%) [11, 12]. Huo et al. [12] also demonstrated that the mutation frequency in the ubiquitin ligase FBXW7 is almost four times higher in African American breast cancer patients (4.2%) than in White patients (1.2%). Furthermore, African American patients show a higher mutation frequency of BRCA1 (10.2%) and BRCA2 (5.7%) tumor suppressor genes comparing to European non-Ashkenazi Jews White patients (BRCA1: 6.9%, BRCA2: 5.2%) [9, 10]. In contrast, mutations in the catalytic subunit of the Alpha isoform of the Phosphatidylinositol 4,5-Bisphosphate 3-Kinase (PIK3CA) were rarer in African American patients than in White breast cancer patients (20% vs 34%). This difference was even more significant between European White patients (36%) and a cohort of Nigerian breast cancer patients (17%) [13]. In the same study [13], Pitt et al. also determined a significantly lower mutation rate of Cadherin 1 (CDH1) in Nigerian patients (0.8%) and TCGA African American patients (6.4%) as compared to White patients (16.2%).

Besides the mutation rates, the frequency of the DNA copy number alterations has been recently analyzed [12]. It was shown that retinoblastoma protein 1 (RB1), a cell cycle suppressor and the CUB And Sushi Multiple Domains 1 (CSMD1), a tumor suppressor that control cell proliferation, invasion, and migration, are more frequently deleted in Black/African American breast cancer patients (14.5% and 8.6%, respectively) as compared to White patients (8.7% and 4.1%, respectively). Conversely, MYC and Cyclin E1, critical activators of the cell cycle, are more frequently amplified in Black/African American breast cancer patients (30.9% and 9.2%, respectively) than in White patients (20.4% and 3.6%). Together, accumulating clinical and genomics data reveal unique molecular features that may contribute to survival disparity in breast cancer. As summarized in Table 1, the majority of genes that are differentially altered in White and African American breast cancer patients play critical functions in cell proliferation and survival. Meanwhile, most of those genes, including TP53, BRCA1/2, FBXW7, RB1, CDH1, and CSMD1, are tumor suppressors lost due to the inactivating mutations or deletions. The discovery of race-specific and therapeutically actionable targets to decrease the mortality in African American breast cancer patients remains a challenge.

To address this unmet medical need, we performed a systematic analysis of clinical outcomes and gene expression determined for the TCGA PanCancer cohorts of White and African American breast cancer patients. We have identified 32 genes as potential targets to decrease the mortality of African American breast cancer patients. The mitogen-activated protein kinase 3 (MKK3) appeared among the proteins with the most dramatic impact on the survival of African American TNBC patients. We determined that MKK3 promotes TNBC tumorigenesis in African American but not White or Asian patients, and its overexpression leads to the activation of the transcriptional program of major tumor driver MYC.

Together, our data revealed multiple proteins as new promising targets for therapeutic intervention in breast cancer African American patients. As one example, we showed that MKK3 has critical oncogenic functions and promotes TNBC tumorigenesis in African Americans through the activation of the MYC program. The discovery of small-molecule inhibitors to control MKK3 signaling may provide a new therapeutic strategy to decrease mortality in African American TNBC patients.

## Methods

### Breast cancer patient cohort

**Fig. 1:**
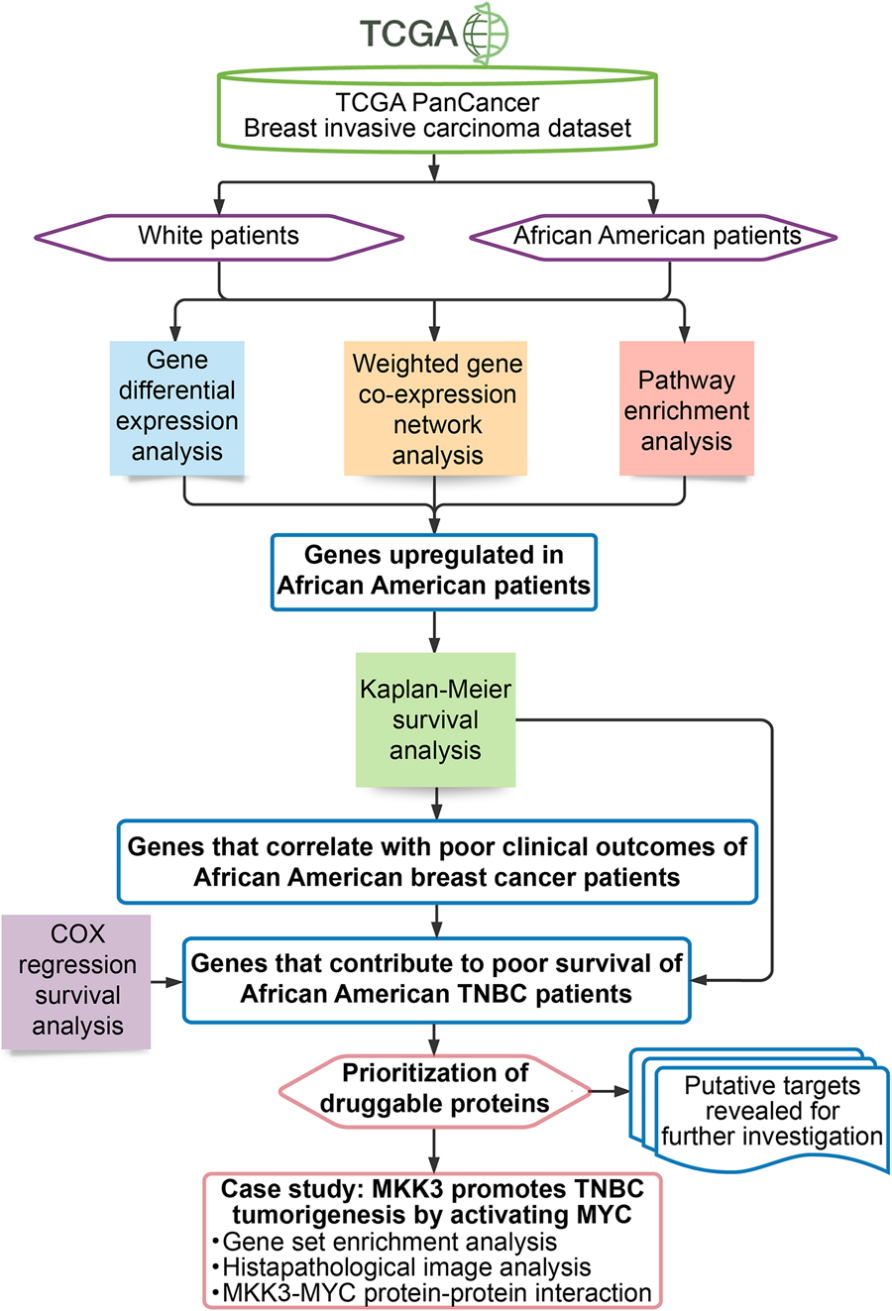
General workflow of the data analysis performed to uncover potentially druggable genes that can contribute in poor clinical outcomes of Black/African American breast cancer patients

In this study, the clinical and genomics data from the Breast Invasive Carcinoma TCGA PanCancer cohorts [14] that consists of a total of 1055 female patients with determined DNA copy-number and mRNA expression were analyzed (Fig. 1). The gene RNA expression, DNA copy number, and breast cancer patient survival data were obtained from the NCI Genomics Data Commons (GDC) [15]. The dataset included samples from 729 White patients (69% of all samples) and 178 samples from Black or African American (BAA) patients (17%), as well as 60 Asian patient samples (6%), and 88 samples (8%) from patients with unspecified race. The breast cancer subtype annotations were added based on the original publication [14].

### Differential expression

The subset of 17,211 protein-coding genes was identified based on the HUGO Gene Nomenclature Committee (HGNC) annotations [16]. The DNA amplifications or deletions were determined based on the GISTIC 2.0 scores (2-amplification, -2-homozygous deletion) [17]. For the differential expression analysis, the TCGA RNA-seqV2 expression data (EBPlusPlusAdjustPANCAN_IlluminaHiSeq_RNASeqV2.geneExp.tsv; http://api.gdc.cancer.gov/data/3586c0da-64d0-4b74-a449-5ff4d9136611) were used. For each gene, the log2 fold change was calculated as $log2 \,fold \,change = \mu_{BAA} - \mu_{WT}$, where *µ*_BAA_ and *µ*_WT_ are the mean values of the log2 (x + 1)-transformed gene expression obtained for the Black/African American and White patient cohorts, respectively. The p-values were calculated with the Wilcoxon test. The false discovery rate adjusted q-values were calculated with the Benjamini–Hochberg procedure [18].

The mRNA overexpression was determined based on the z-scores. First, the average (µ) and standard deviation (σ) values were calculated for the samples in which gene is diploid. Then, the z-score was calculated as (τ − µ)/σ, were τ is the gene mRNA expression in the sample. Z-score > 2 and z-score < -2 indicates gene overexpression or underexpression, respectively.

The signed weighted gene co-expression network was constructed for 5256 genes differentially expressed in Black/African American and White breast cancer patients using the WGCNA R package [19]. Pearson correlation coefficients between the expression of all gene pairs were also calculated and used to construct the adjacency correlation matrix and the topological overlap matrix (TOM). The optimal value of the soft threshold power β = 11 was selected using the pickSoftThreshold function to maintain the scale-free topology and sufficient node connectivity [20]. The hierarchical clustering of genes was performed based on the TOP matrix using the average agglomeration method implemented in the flashClust function [21]. The gene modules were identified using the dynamic tree cut method [22]. Specifically, the cutreeDynamic R function was used with the minModuleSize = 100 and method = ”tree” options.

### Survival analysis

The Kaplan–Meier survival curves and the logrank p-values have been calculated using the Lifelines python package. The mean survival time (MST) values were calculated with the Lifelines package based on the area under the survival curve. The COX regression analysis has been performed using the fit proportional hazards regression model function coxph from the Survival R package.

### Enrichment analysis

The disease association and pathway enrichment analysis were performed using the DisGENet [23], KEGG [24], and Reactome [25] datasets. The p-values were calculated with the Fisher Exact test using 17,211 protein-coding genes as the reference set. The false discovery rate adjusted q-values were calculated with the Benjamini–Hochberg procedure [18]. The gene set enrichment analysis (GSEA) was performed using the GSEA program [26]. The High and Low phenotypes were defined as the 10% of samples with the highest and the lowest gene expression, respectively. The GSEA curves were rebuilt using the GseaPy python package.

### The breast cancer histological image analysis

#### Fully-convolutional model training

To extract tumor features we used our established standard 16-layer VGG fully-convolutional neural network (VGG16-FCN8) constructed using ImageNet [27] pre-trained weights as described previously [28]. We have previously shown that for this particular dataset, the VGG-16 FCN-8 architecture shows more favorable model convergence and fitting properties than the deeper and more complex DenseNet architecture [29]. Using this particular architecture and number of layers enabled us to leverage the publicly available pre-trained weights, hence improving accuracy [28, 29].

The model is trained to classify pixels into one of five classes: tumor (including DCIS), stroma, tumor-infiltrating lymphocytes (including plasma cells and mixed inflammatory infiltrates), necrosis or debris, and others. Regions of interest were divided into 800 × 800 pixel tiles that are overlapping, where the amount of overlap increased for smaller regions of interest to create a balanced training dataset. Random cropping of 768 × 768 pixel regions was used as a data augmentation strategy to improve robustness during training. The model was trained on 4 GPUs with a per-GPU batch size of 4 tiles (16 tiles per batch) using data parallelization and gradient averaging. Adap optimizer was used with a starting learning rate of 1e−5. The loss function used is weighted categorical cross-entropy, where the weight associated with each region class, Wc, is calculated using the equation:

$$ W_{c} = \left\{ \begin{aligned} 0:\quad \quad {\kern 1pt} \quad {\text{if }}c = 0 \hfill \\ 1 - \frac{{N_{c} }}{N}:\quad {\text{if }}c > 0 \hfill \\ \end{aligned} \right. $$

where *N* is the total number of pixels and *Nc* is the total number of pixels belonging to region class *c.*

#### Fully-convolutional model inference

We used whole-slide images (WSI) formalin-fixed paraffin-embedded hematoxylin and eosin-stained slides from the TCGA cohort. The analysis was focused on WSIs from African-American patients with triple-negative breast cancer, and limited to infiltrating ductal histologic subtype (determined using TCGA clinical records). The focus on infiltrating ductal subtype is for pragmatic reasons since the fully-convolutional model has been trained and optimized on this histologic subset. Only one diagnostic slide was used per patient (“-DX” designation in TCGA) and only WSIs scanned at 40× were used in the analysis. The analysis was performed at scan magnification.

Analysis regions were chosen semi-automatically and constituted the main tumor bulk within a WSI. A low-resolution RGB image of the slide (at 0.3–0.5 x) was loaded and converted to the Hue-Saturation-Intensity (HSI) space. Default thresholds for each of the HSI channels were manually adjusted for each slide to capture the majority or entirety of the tumor within the slide. This region of interest was divided into non-overlapping 1024 × 1024 pixel tiles and fed into the trained FCN-8 model after color normalization using the Reinhard method [30]. The Reinhard normalization used target statistics derived from the RGB image corresponding to the mask called *“TCGA*-*A2*-*A3XS*-*DX1_xmin21421_ymin37486_.png”* [28].

#### Feature extraction of tumor nests

A total of nine features (four global and five local) were derived from the slides. “Local” features are those features derived from each individual tumor nest (defined as a coherent collection of carcinoma cells) and are averaged to get slide-level features. The global features were: tumor-to-stroma ratio, stromal tumor-infiltrating lymphocyte score, necrosis-to-tumor ratio, and the number of tumor nests, normalized for the area of the region of interest (i.e. “per pixel”). Local features included area and shape descriptors for each tumor nest.

#### Histologic-genomic correlation

Histological descriptors were compared against gene expression data derived from the same patients in the TCGA cohort. Spearman correlation coefficient was used and the Benjamini–Hochberg adjustment was used for multiple hypothesis testing.

## Results

### Differential gene expression in African American and White breast cancer patients

**Fig. 2:**
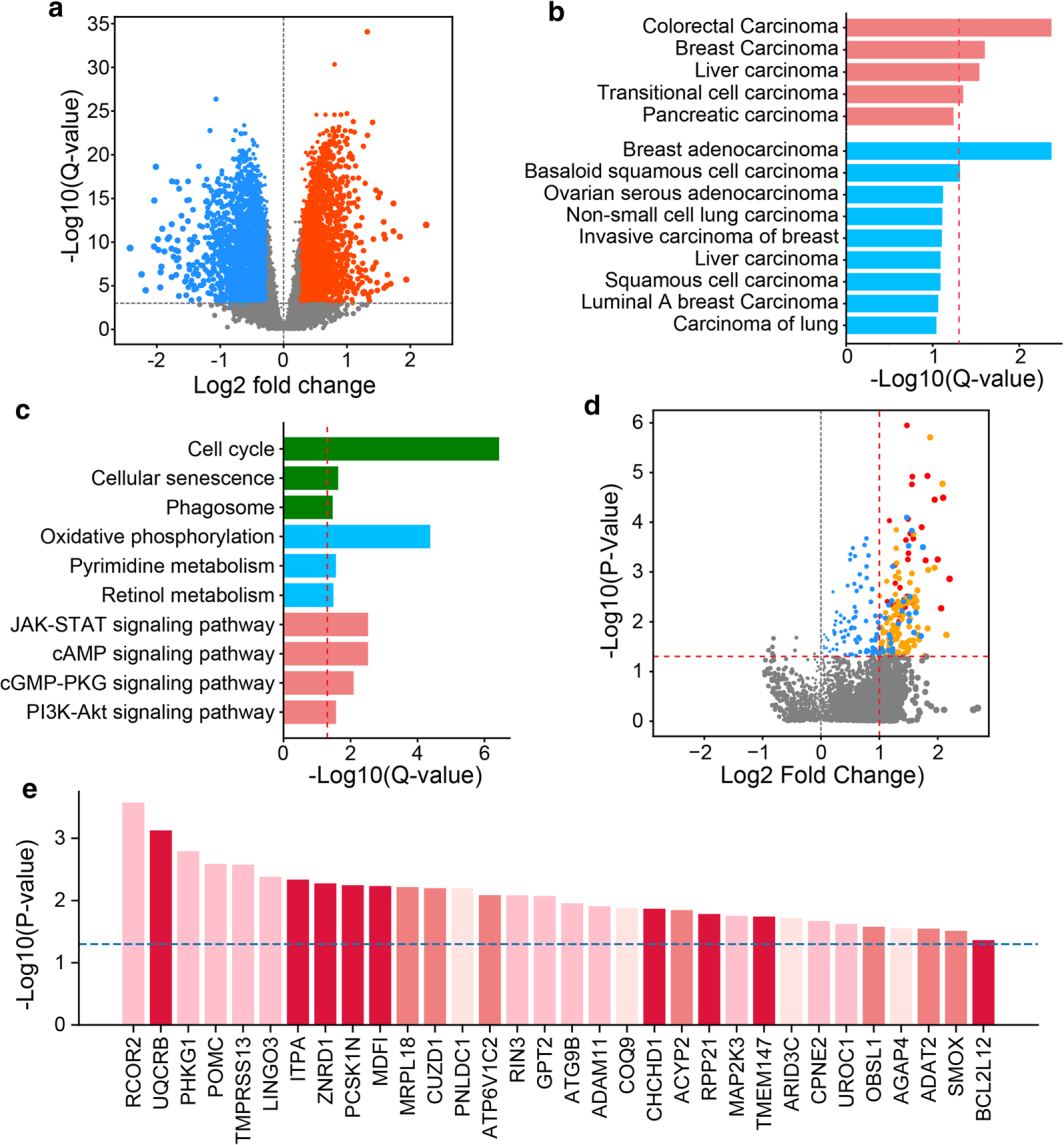
Differential expression and breast cancer patient survival analysis. **a** Differential expression analysis revealed 2501 genes downregulated (blue) and 2767 genes upregulated (orange) in Black/African American (BAA) patients as compared to White patients. **b** Enrichment analysis of the disease-associated datasets revealed strong association (p-value < 0.01 and q-value < 0.1) between different cancer types and genes either upregulated (red) or downregulated (blue) in BAA breast cancer patients as compared to White patients. The dash-line indicates the q-value < 0.05 cut-off for the most statistically significant enrichment. **c** Enrichment analysis revealed the biological processes (green), metabolic (blue), and signaling (red) pathways associated with genes overexpressed in at least 5% of BAA breast cancer patients and upregulated as compared to White patients. **d** The volcano plot shows the association of high gene expression with decreased survival of BAA breast cancer patients. The fold change was calculated as the ratio of mean survival time of patients with normal gene expression to mean survival time of patients with gene overexpression. Overexpression of 257 genes (blue) correlates with decreased BAA patient survival (log-rank p_BAA_-values < 0.05). Overexpression of 147 genes was not correlated with reduced survival of White patients with the p_WT_-value > 0.1 (orange). The most significant difference between BAA and White breast cancer patient survival was observed for overexpression of 32 genes highlighted in red (p_BAA_-values < 0.01, p_WT_-value > 0.1, P_WT/BAA_-value < 0.05, MST_WT_/MST_BAA_ > 2). **e** The top-32 genes with the most BBA patient-specific contribution in decreased survival are shown. Y-axis indicates the log-rank p-values calculated for BAA and White patients with gene overexpression. The blue line indicates the p-value cut-off of 0.05. The bars are colored based on the frequency of gene overexpression in BAA breast cancer patients. Light-pink: 5–6%, pink: 6–10%, light-red: 10–15%, dark-red: > 15%

To determine the differences between gene expression in White and Black/African American breast cancers, we have performed the differential expression (DE) analysis for a total of 17,211 protein-coding genes. We found that 7195 genes showed statistically significant differences in expression between White and Black/African American cohorts, as determined with the Wilcoxon test p-values adjusted for the false discovery rate (q-value < 0.001, Additional file 1: Table S1). To increase the stringency of the analysis, we further prioritized 5268 genes with q-values < 0.001 and at least 20% difference in the mRNA expression in White and Black/African American patients (Fig. 2a). Among those genes, expression of 2501 genes was decreased in Black/African American patients, as compared to White breast cancer patients (BAA_low_ gene set). In contrast, the expression of 2767 genes was significantly higher in the Black/African Americans cohort than in White patients (BAA_high_ gene set). These data indicate that White and Black/African American breast cancer patients have very different genomic backgrounds with over 30% of the protein-coding genes expressed differently in these two patient cohorts.

### Cancer association and pathway enrichment analysis

To determine whether the differentially expressed genes are associated with the regulation of oncogenic processes, we performed the enrichment analysis. First, using the carcinoma-associated gene sets defined in DisGeNET database [23], we found that both, BAA_high_ and BAA_low_ gene sets are significantly enriched (p-value < 0.01, q-value < 0.1) in genes associated with different cancer types, including colon (q-value = 0.006), liver (q-value = 0.036 for BAA_high_ and q-value = 0.087 for BAA_low_), pancreatic (q-value = 0.068), and lung cancers (q-value = 0.091) (Fig. 2b). Moreover, genes associated with breast carcinoma were among the most significantly overrepresented genes in both, BAA_high_ (p-value < 0.001, q-value < 0.031) and BAA_low_ sets (p-value < 0.001, q-value = 0.004, Fig. 2b). 2567 out of 2767 BAA_high_ genes (92%) are overexpressed in at least 5% of Black/African American breast cancer patients, supporting their potential roles in breast carcinogenesis.

Then, we sought to determine specific biological programs associated with identified differentially expressed genes. Through the enrichment analysis of signaling and metabolic pathways defined in the KEGG database [24], we found that overexpressed BAA_high_ genes (BAA_OVR_ genes) showed the enrichment in genes associated with several major oncogenic pathways. The most significant enrichment (p-value ≪ 0.001, q-value ≪ 0.001) was observed for the cell cycle-associated genes. Furthermore, genes involved in senescence, phagosome maturation, JAK-STAT, cAMP, cGMP-PKG, and PIK3-AKT signaling pathways, retinol and pyrimidine metabolism, and oxidative phosphorylation (Fig. 2c) were significantly overrepresented in the BAA_OVR_ genes (p-value < 0.001, q-value < 0.05). Thus, overexpression of genes upregulated in Black/African American patients may promote breast cancer development and progression through the dysregulation of multiple oncogenic processes.

To identify functional modules of co-regulated genes we applied the weighted gene co-expression network analysis (WGCNA) [19, 20]. The WGCNA performed for all 5268 BAA_high_ and BAA_low_ genes revealed 12 distinct modules of significantly co-expressed genes (Additional file 1: Table S2, Additional file 2: Figure S1). The “pink” (332 genes), “black” (355 genes), “cyan” (846 genes), “red” (368 genes), “green” (431 genes), and “magenta” (292 genes) modules were comprised almost completely by BAA_high_ genes. The “yellow” (521 genes), “blue” (798 genes), “brown” (648 genes), “purple” (174 genes), “greenyellow” (164 genes), and “tan” (143 genes) modules included mostly BAA_low_ genes. To uncover the biological pathways associated with individual modules, we performed the enrichment analysis using the gene sets defined in the KEGG database [24] (Additional file 1: Table S3; Additional file 2: Figure S2). We found that five modules were more than tenfold overrepresented by genes involved in pathways defined in the KEGG database as compared to the reference human genome. Among all modules, the most significant enrichment was determined for the “pink” module, which appeared to be overrepresented (overrepresentation fold, OVF = 21.08, q-value = 8.78 × 10^−26^) in the cell cycle regulating genes (Additional file 1: Table S3). An equally high overrepresentation (OVF = 20.83, q-value = 2.38 × 10^−6^) was determined for the “magenta” module that was enriched in genes that control primary immunodeficiency, including ADA, CD19, CD79A, IGLL1, TAP1, TAP2, TNFRSF13C, and ZAP70. The “green” module appeared to be enriched in genes involved in oxidative phosphorylation (OVF = 12.5, q-value = 8.50 × 10^−14^), ribosome (OVF = 12.43, q-value = 4.83 × 10^−12^), and genes involved in neurodegenerative disorders, such as the Parkinson’s disease (PD) (OVF = 11.31, q-value = 4.83 × 10^−12^). Notably, multiple studies have suggested that the development of PD and cancer, including breast cancer, can progress through the same genes and molecular mechanisms [31–33]. In contrast to “pink”, “magenta”, and “green” modules, the “tan” and “greenyellow” modules are comprised of genes with higher expression in White breast cancer patients as compared to Black/African American patients. We found that the “tan” module is enriched in genes involved in extracellular matrix receptor interactions (OVF = 15.31, q-value = 2.39 × 10^−6^). The “greenyellow” module appeared to be overrepresented in the ATP-binding cassette (ABC) transporters (OVF = 19.77, q-value = 4.85 × 10^−5^), and genes that control tyrosine metabolism (OVF = 10.15, q-value = 2.98 × 10^−2^) and the complement and coagulation cascades (OVF = 14.37, q-value = 4.85 × 10^−5^). The role of the ATP-binding cassette (ABC) transporters in tumorigenesis of White breast cancer patients is further supported by the enrichment of the “brown” module in basal transcription factors, including the ATP-binding cassette subfamily members ABCA9, ABCC9, ABCG2, ABCB1, ABCA6, and ABCA8. Previous studies have demonstrated the association of ATP-binding cassette transporters with breast cancer aggressiveness and reduced survival of breast cancer patients [34, 35]. We also noticed that the “blue” module is enriched (OVF = 6.21, q-value = 0.036) in genes that control sphingolipid metabolism that play critical functions in cancer growth and progression [36]. Furthermore, the “purple” module appeared to be enriched in genes that are associated with the dilated cardiomyopathy, a known side effect of breast cancer radiotherapy and chemotherapy [37, 38].

### Differential expression and survival disparity

The genes with abnormally high expression may represent putative targets for therapeutic intervention. We used the set of 2567 BAA_OVR_ genes to determine the impact of their overexpression on breast cancer patient survival. We found that overexpression of 257 BAA_OVR_ genes (Additional file 1: Table S4, Group I) correlates with decreased survival of Black/African American patients (p_BAA_-value < 0.05) (Fig. 2d). Furthermore, the overexpression of 174 out of 257 genes (Additional file 1: Table S4, Group II) correlated with more than twofold decreased survival. Among the 174 genes, overexpression of 147 genes (Additional file 1: Table S4, Group III) was associated with the reduced survival of Black/African American patients, but not White patients (p_WT_-value > 0.1, Fig. 2d). This group of genes includes several genes previously linked with breast cancer development and progression. For example, overexpression of protein arginine methyltransferase 1 (PRMT1) has been associated with the methylation of the transcription factor C/EBPα and inhibition of its tumor suppressor function in breast cancer [39]. Interestingly, PRMT1 knockdown was also correlated with decreased EGFR activity and suppressed proliferation of in MDA-MB-468 breast cancer cells that are derived from an African American breast cancer patient [40]. Kinesins KIF1C and KIFC3 promotes breast cancer cell growth and survival and mediate taxane resistance [41, 42]. Syndecan-1 (SDC1) has been linked with the accelerated metastasis of breast cancer to the brain [43]. Meanwhile, our data revealed genes previously not associated with the increased breast cancer progression, providing new opportunities for therapeutic interventions in breast cancer.

**Fig. 3:**
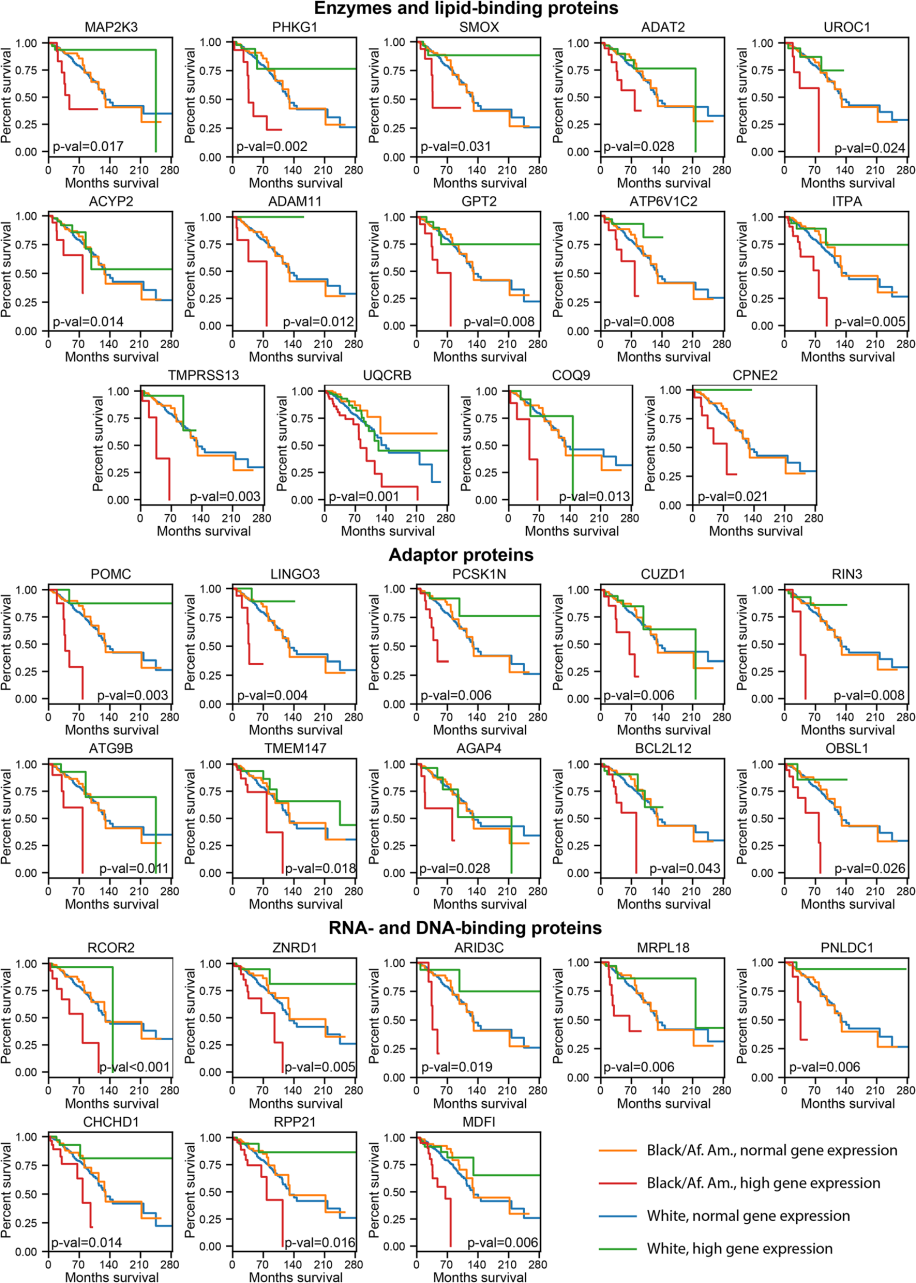
Survival curves for the top-32 genes that contribute to survival disparity between Black/African American and White patients. Orange and red lines indicate the survival of Black/African American breast cancer patients with normal and overexpressed gene levels, respectively. Blue and green lines indicate the survival of White breast cancer patients with normal and overexpressed gene levels, respectively. The log-rank p-values calculated for the survival rates of White and Black/African American patients with gene overexpression are indicated

To further prioritize genes with the most significant contribution to the survival disparity between African American and White patients, we applied more stringent statistical cut-offs: p_BAA_-value < 0.01, p_WT_-value > 0.1, a significant difference between survival time of White and Balck/African American patients with the overexpressed gene (p_WT/BAA_-value < 0.05), and at least twofold decreased mean survival time of Black/African American patients (MST_BAA_) comparing to the MST of White patients (MST_WT_) determined for the samples with the overexpressed gene. Using these parameters, a total of 32 genes with a most significant and race-specific impact on the breast cancer tumorigenesis in Black/African American patients have been prioritized (Additional file 1: Table S4, Group IV, Fig. 2e, and Fig. 3).

### Evaluation of protein druggability for therapeutic discovery

To assess the potential druggability of the top-32 prioritized proteins, we have classified them into three groups based on the protein types (Fig. 3). RCOR2, ZNRD1, ARID3C, MRPL18, PNLDC1, CHCHD1, RPP21, MDFI are either DNA- or RNA-binding proteins. These proteins may represent the most challenging targets for direct interrogation with small molecules or specific antibodies due to their nuclear localization the lack of a defined pocket for a small-molecule binding. POMC, LINGO3, PCSK1N, CUZD1, RIN3, ATG9B, TMEM147, AGAP4, BCL2L12, OBSL1 also lack an enzymatic activity and contribute in breast cancer tumorigenesis acting as adaptors for other proteins. A large area, hydrophobicity, and relatively flat configuration of the protein–protein interaction (PPI) interface surfaces are among the limiting factors for the design and discovery of low molecular weight PPI inhibitors [44]. On the other hand, the growing number of potent cell-permeable inhibitors for PPI discovered over the past decades, including the FDA-approved BCL2 inhibitor venetoclax [45], indicates the PPI druggability for therapeutic discovery [46]. Meanwhile, enzymes and receptors represent the largest class of therapeutic targets [47]. We found that 14 out of 32 proteins belong to protein families known to be druggable by low molecular weight compounds. Specifically, COQ9 and CPNE2 are the lipid-binding proteins with a defined binding site for a lipid molecule that can be targeted by small molecules [48]. Furthermore, 12 proteins belong to different types of enzymes, including a subunit of the ubiquinol-cytochrome c oxidoreductase UQCRB, serine protease TMPRSS13, inosine triphosphate pyrophosphatase ITPA, proton ATPase ATP6V1C2, Alanine aminotransferase GPT2, metalloproteinase ADAM11, acylphosphatase ACYP2, urocanate hydratase UROC1, tRNA-specific adenosine deaminase ADAT2, spermine oxidase SMOX, and two kinases: PHKG1 and MKK3 also known as MAP2K3. The discovery of potent inhibitors for these enzymes may lead to new therapeutic strategies for African American breast cancer patients.

### COX regression survival analysis for TNBC Black/African American patients

The COX regression analysis is a widely used approach to identify predictive biomarkers of poor clinical outcomes [49, 50]. We applied the COX regression analysis to determine the overall impact of the prioritized genes on clinical outcomes of Black/African American patients specifically with the triple-negative breast cancer subtype. First, we built the univariate COX regression models to determine the hazard ratios and significance for each of the 32 prioritized genes. We found that for each gene the Hazard ratio values (HR) were higher than 1 indicating a positive correlation between gene expression and decreased patient survival (Additional file 1: Table S5). This result is consistent with the Kaplan–Meier analysis performed for all breast cancer subtypes (Fig. 3). Eight out of 32 genes demonstrated highly significant correlation with poor clinical outcomes with the Hazard ratio (HR) > 2 and the p-values ≤ 0.05, including ACYP2, ADAT2, AGAP4, CHCHD1, MKK3, MRPL18, RPP21, and ZNRD1 (Additional file 1: Table S5; Additional file 2: Figure S3).

To evaluate the combined effect of these 8 genes on the clinical outcomes of TNBC Black/African American patients, we built a multivariate COX regression model. The resulting Model 1 demonstrated a high concordance index (c-index = 0.93) and statistical significance (p-value = 1 × 10^−4^), indicating the satisfactory prognostic ability of the model (Additional file 1: Table S6). The detailed evaluation of the model revealed that expression of MKK3 (HR = 27.98, p-value = 0.002), AGAP4 (HR = 1.73, p-value = 0.017), and ACYP2 (HR = 1.30, p-value = 0.04) made the most significant contribution to the model. To determine if MKK3, AGAP4, and ACYP2 can be used as markers for poor clinical outcomes, we built another model based on these three genes only. The resulting Model 2 (Additional file 1: Table S6) was characterized by an equally high c-index of 0.91 and improved statistical significance (p-value = 2 × 10^−5^) as compared to the 8-parameter model. We noticed that in both Model 1 and Model 2 the highest HR value was obtained for MKK3, suggesting its significance for clinical outcomes of Black/African American TNBC patients.

### MKK3 overexpression promotes triple-negative breast cancer in African American patients

**Fig. 4:**
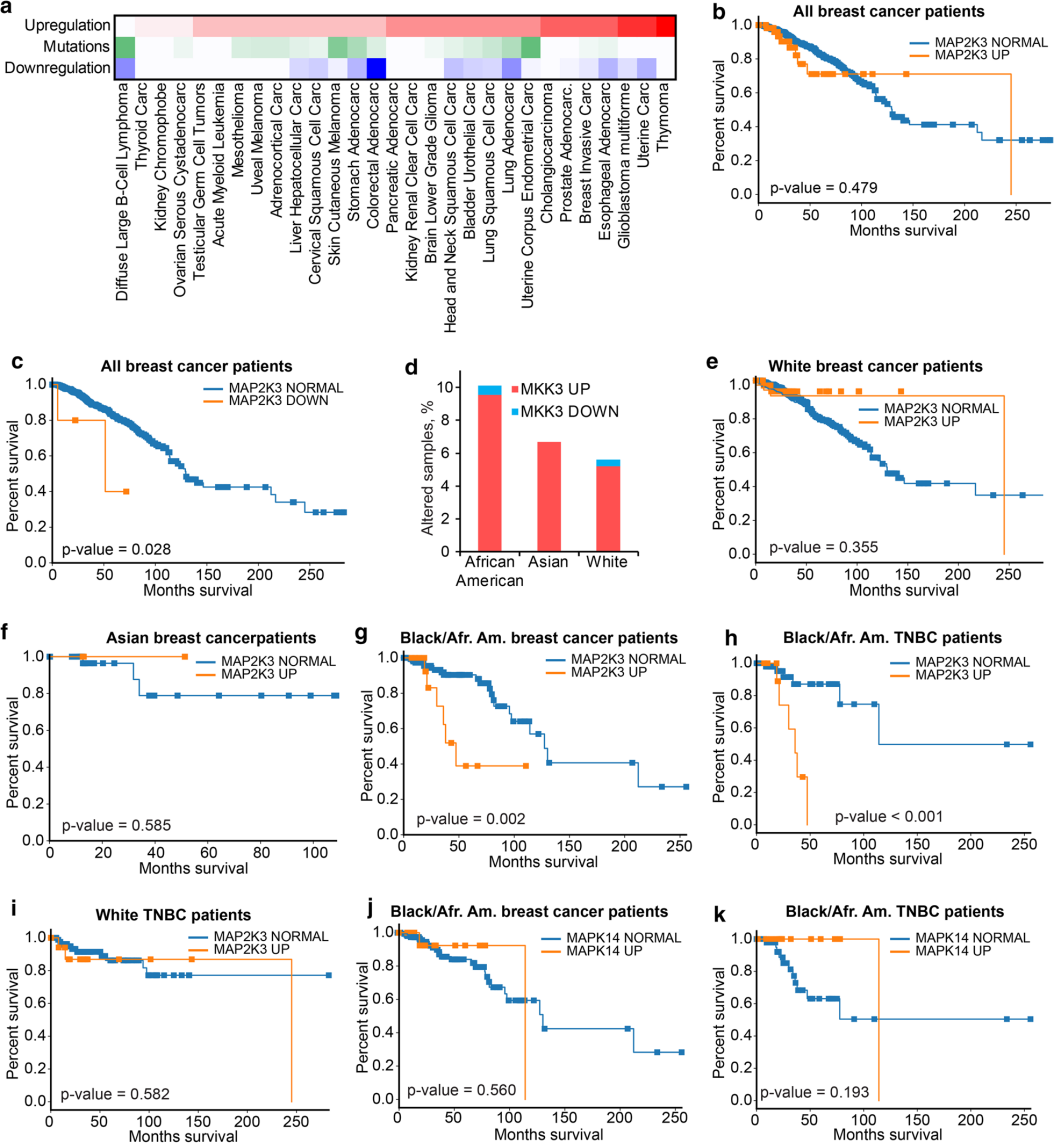
MKK3 upregulation correlates with poor survival of Black or African American breast cancer patients. **a** The heatmap shows the frequency of genomic alterations of MKK3 in different cancer types. The percent of samples with MKK3 overexpression or amplification is shown with red color ranging from the smallest (light color) to the highest (strong color) values. The frequency of MKK3 mutations is indicated with the gradient of green color. MKK3 underexpression or deletion is highlighted in blue color. **b** MKK3 upregulation does not correlate with poor survival of the pooled cohort of breast cancer patients. **c** MKK3 deletions observed in 0.5% of breast cancer patient correlates with decreased patient survival. **d** Distribution of MKK3 upregulation in Black/African American, Asian, and White cohorts of breast cancer patients. **e** The upregulation of MKK3 does not correlate with decreased survival of White breast cancer patients. **f** The upregulation of MKK3 does not correlate with decreased survival of Asian breast cancer patients. **g** The upregulation of MKK3 correlates with poor survival of Black/African American breast cancer patients. **h** The upregulation of MKK3 correlates with decreased survival of Black/African American triple-negative breast cancer patients. **i** The upregulation of MKK3 does not correlate with poor survival of White TNBC patients. **j** Upregulation of p38 (MAPK14) does not correlate with poor survival of Black/African American breast patients. **k** In contrast to MKK3, the upregulation of MAPK14 does not correlate with decreased survival of TNBC Black/African American patients

MKK3 is frequently altered in different cancers and recent studies have suggested that MKK3 may contribute in tumorigenesis in multiple cancer types [51–55]. Analysis of the TCGA PanCancer datasets indicates that MKK3 is mutated in 5% of uterine carcinoma, 5% of B-cell lymphoma, and 4% of skin melanoma patients. MKK3 is homozygously deleted in 6% of colon cancer patients. On the other hand, MKK3 is either overexpressed or amplified in 3 to 8% of patients in the vast majority of cancers, including thymoma (8%), glioblastoma multiform (7%), and breast invasive carcinoma (6%) (Fig. 4a, Additional file 1: Table S7). Furthermore, MKK3 overexpression can be triggered by TP53 mutations [56], that can link MKK3 to TP53-dependent cancers, such as breast cancer, particularly in African American patients.

The evaluation of the overall survival data for the pooled dataset of 1055 samples revealed no correlation between the MKK3 overexpression and patient survival (p = 0.479, Fig. 4b, Additional file 1: Table S8). Instead, five patients with lost MKK3 demonstrated decreased survival comparing to patients with normal MKK3 (p = 0.028, Fig. 4c). This observation is consistent with a previous report that MKK3 may play a tumor-suppressive role in breast cancer [57]. Meanwhile, we found that MKK3 is the most frequently overexpressed in the Black/African American cohort (9.6%) (Fig. 4d). In the Asian and White breast cancer patients, MKK3 is overexpressed in 6.7% and 5.2%, respectively. Conversely, MKK3 downregulation is not frequent in breast cancer patients. MKK3 is not underexpressed or deleted in the Asian cohort, and it was deleted in 3 White patients (0.4%), 1 Black patient (0.6%), and 1 patient with the unspecified race (1.1%). In agreement with the genomic status of MKK3, its upregulation does not correlate with poor survival of White (p = 0.355, Fig. 4e) nor Asian (p = 0.585, Fig. 4f) patients. In contrast, a strong decrease in patient survival (p = 0.002) was observed for the Black/African American cohort (Fig. 4g).

The analysis of the histological subtypes of breast patients indicates, that the majority of Black/African American patients in the breast cancer TCGA PanCancer cohort (N = 178) have either basal-like/triple-negative (TNBC) (63 patients) or Luminal A (61 patients) breast cancer. The number of patients with Luminal B, HER2, and Normal breast cancers was 28, 16, and 10, respectively. Surprisingly, MKK3 was upregulated in only one patient with the Luminal A breast cancer. In contrast, MKK3 was overexpressed in 19% of Black/African American TNBC patients.

Similar to the combined set of breast cancer samples of all subtypes, the MKK3 overexpression correlates with poor survival of TNBC Black/African American patients (p < 0.001, Fig. 4h), but not White patients (p = 0.582, Fig. 4i). Moreover, through a systematic analysis of all breast cancer subtypes in all racial groups of patients, we have determined that MKK3 upregulation correlates uniquely with the poor survival of Black/African American patients specifically with the TNBC, and not with any other race or other breast cancer subtypes (Additional file 1: Table S9).

### MKK3 promotes TNBC through a p38-distinct mechanism

MKK3 is the main activator of its only known substrate p38 which plays a key role in the induction of apoptosis and regulation of inflammation in response to extracellular stress [58, 59]. It can be expected that the poor survival of Black/African American patients is also associated with p38 activation. p38 (encoded by the MAPK14 gene) is amplified or overexpressed in 9.5% of the Black/African American breast cancer patients. However, in contrast to MKK3, p38 upregulation does not correlate with decreases survival of Black/African American patients neither for all breast cancer subtypes (p = 0.986) (Fig. 4j) nor specifically for the TNBC (p = 0.193, Fig. 4k). These data suggest a p38-distinct role for MKK3 in TNBC tumorigenesis. These results are further supported by the recent discovery of MKK3 as a hub protein in the PPI network determined for cancer-associated proteins [60, 61]. It was shown that besides p38, MKK3 can bind to multiple other proteins, including several drivers of breast cancer, such as CDK4, AURKA, FGFR4, EPHA2, and MYC [60].

### MKK3 activates MYC transcriptional program in TNBC African American patients

To uncover the molecular bases underlying the decreased survival of TNBC Black/African American patients, we performed the Gene Set Enrichment Analysis (GSEA) [62] against 50 hallmark sets of genes that define signatures of specific biological state or process [26, 63, 64] (Additional file 1: Table S10). Only five gene sets showed significant enrichment in samples with upregulated MKK3 expression (p-value < 0.05 and FDR < 25%), including genes involved in unfolded protein response, mTORC1 signaling, response to the UV irradiation, and two sets of MYC target genes [64, 65] (Additional file 1: Table S10).

**Fig. 5:**
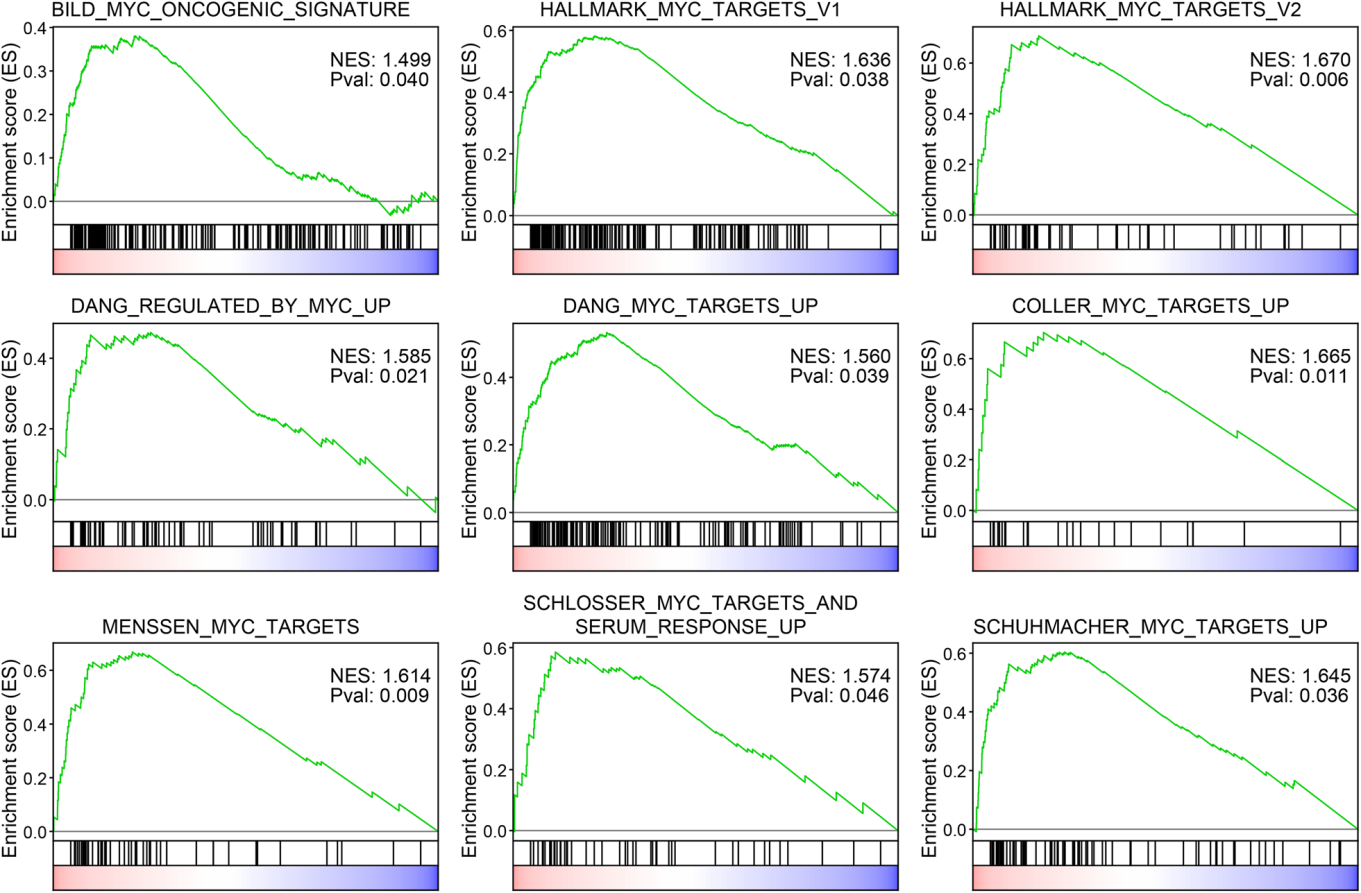
The tumor samples from Black/African American TNBC patients with a high level of MKK3 are enriched in MYC-target genes. The Gene Set Enrichment Analysis (GSEA) curves are shown for nine gene sets that demonstrated the most significant enrichment in samples with high MKK3 expression (red color on the heatmap) comparing to the low MKK3 expression (blue color). The p-values and Normalized Enrichment Scores (NES) are indicated

To further increase the confidence in the MKK3-MYC functional association, we have expanded the GSEA analysis using 16 more sets of MYC-upregulated genes independently defined in different studies (Additional file 1: Table S11). We found that 17 out of 18 tested MYC-target gene sets demonstrate the enrichment in Black/African American TNBC samples with a high level of MKK3. Furthermore, 9 out of 18 sets showed a statistically significant enrichment with the p-value < 0.05, including the MYC oncogenic signature genes derived from the DNA microarray analysis of the breast cancer cells (p = 0.040, FDR = 5.6%, normalized enrichment score, NES = 1.5) (Fig. 5). Meanwhile, no enrichment in MYC-target genes was found for the samples with upregulated p38, further supporting p38-distinct functions of MKK3 in Black/African American TNBC patients.

As a master regulator, MYC controls multiple oncogenic programs. We sought to determine biological pathways that could be dysregulated specifically in response to MKK3-mediated MYC MYC-activation. Based on the GSEA analysis for each MYC-dependent gene set we determined a total of 323 core genes that contribute the most to the enrichment. The pathway overrepresentation analysis revealed a strong association of 222 of MYC-regulated genes enriched in patients with overexpressed MKK3 with 117 signaling and metabolomic pathways defined in the Reactome database (q-value < 0.01, at least twofold overrepresentation as compared to the reference human genome). We found that the cell cycle and RNA metabolism and processing appeared among the pathway with the most significant overrepresentation in MKK3-MYC core enrichment genes (Additional file 1: Table S12). Interestingly, the REACTOME_DEASES gene set also appeared within the top-10 the most overrepresented pathways suggesting the pathological functions for the genes upregulated through the MKK3-MYC interaction.

**Fig. 6:**
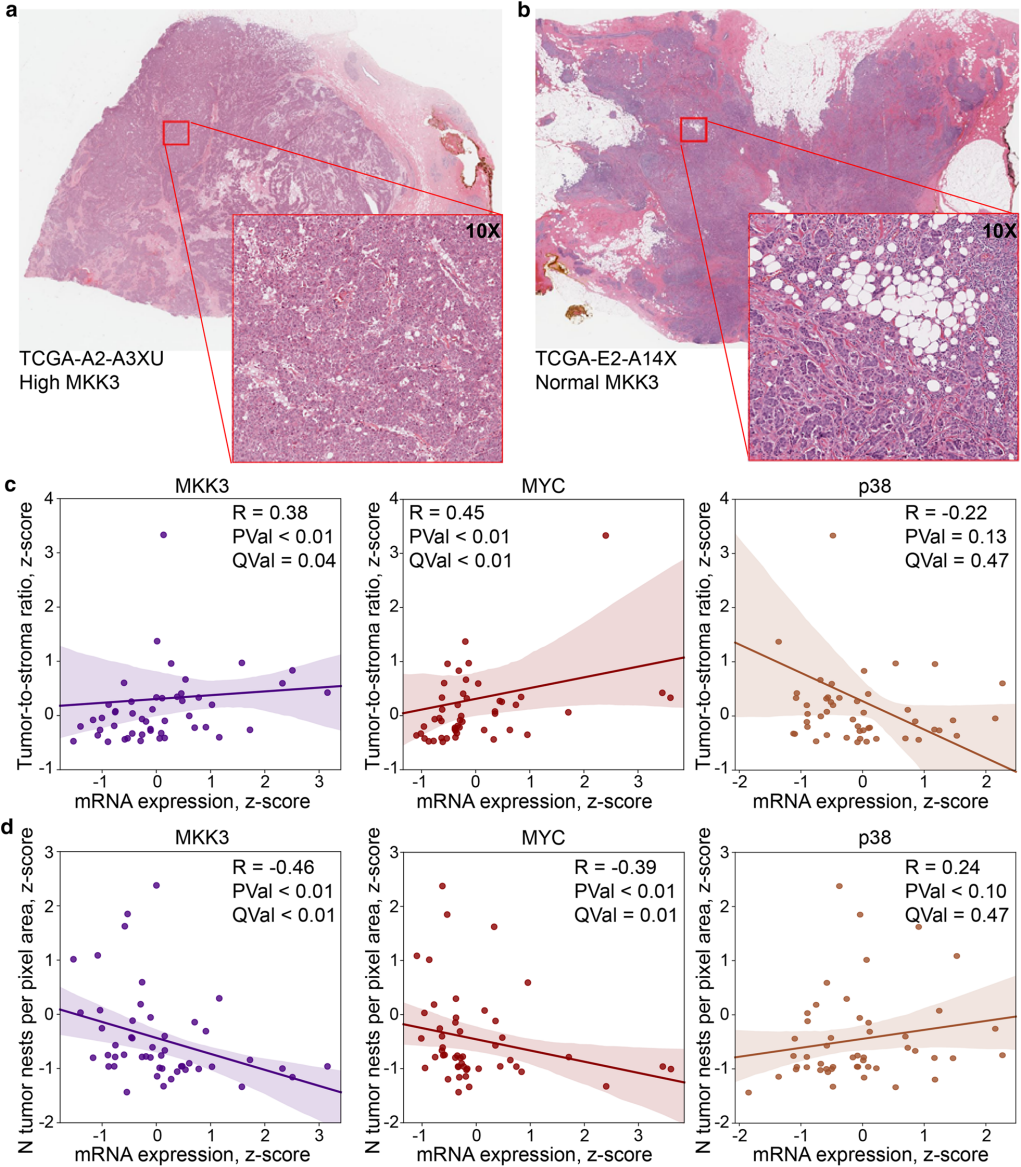
MKK3 increases the tumor aggressiveness in Black/African American patients with triple-negative breast cancer. **a** Representative histological image of the basal-like BRCA tumor from a Black/African American patient (TCGA patient id: TCGA-A2-A3XU) with the overexpressed MKK3. **b** Representative histological image of the basal-like BRCA tumor from a Black/African American patient (TCGA patient id: TCGA-E2-A14X) with a normal level of MKK3 expression. **c** The tumor to stroma ratio calculated for basal-like BRCA tumor samples from Black/African American patients correlates positively with the expression of MKK3 and MYC and negatively with the expression of p38. The Spearman correlation coefficients (R), p-values, and the false discovery rate adjusted q-values are indicated. **d** MKK3 and MYC expression, but not p38 expression, correlates positively with the increased size and decreased the number of tumor nests per unit area (i.e. “fusion” into large infiltrative nests with less intervening stroma). The Spearman correlation coefficients (R), p-values, and the false discovery rate adjusted q-values are indicated

To support the clinical significance of MKK3 as a mediator of TNBC pathology, we performed a quantitative analysis of the histopathological images from Black/African American TNBC patients (Fig. 6a, b). We found that the high level of MKK3 expression is associated with the increased overall tumor to stroma ratio (Spearman R = 0.38, p-value < 0.01, q-value = 0.04, Fig. 6c), and fewer discrete tumor “nests” (Spearman R = −0.46, p-value < 0.01, q-value < 0.01, Fig. 6d). Note that the smaller number of discrete nests is, in this phenotype, a consequence of their larger size, causing less intervening stroma and apparent “fusion” into large invasive tumors (Fig. 6a versus b). Similar trends have been observed for MYC. The elevation of MYC expression leads to increased overall tumor-to-stroma ratio (Spearman R = 0.45, p-value < 0.01, q-value < 0.01, Fig. 6c), as well as fewer discrete tumor nests (Spearman R = −0.39, p-value < 0.01, q-value = 0.01, Fig. 6c), which are, individually, significantly larger in size (Spearman R = 0.42, p-value < 0.01, q-value < 0.01, not shown). Unlike MKK3 and MYC, p38 upregulation does not correlate with either the overall tumor-to-stroma ratio (Spearman R = −0.22, p-value = 0.13, q-value = 0.47, Fig. 6b) or the number of discrete tumor nests (Spearman R = 0.24, p-value = 0.10, q-value = 0.47, Fig. 6c). Moreover, the observed trends, although not statistically significant, were opposite compared to trends determined for MKK3 and MYC. Together, these data suggest a critical role of MKK3 in promoting the TNBC tumorigenesis in African American patients and its strong association with the activation of the MYC program.

**Fig. 7:**
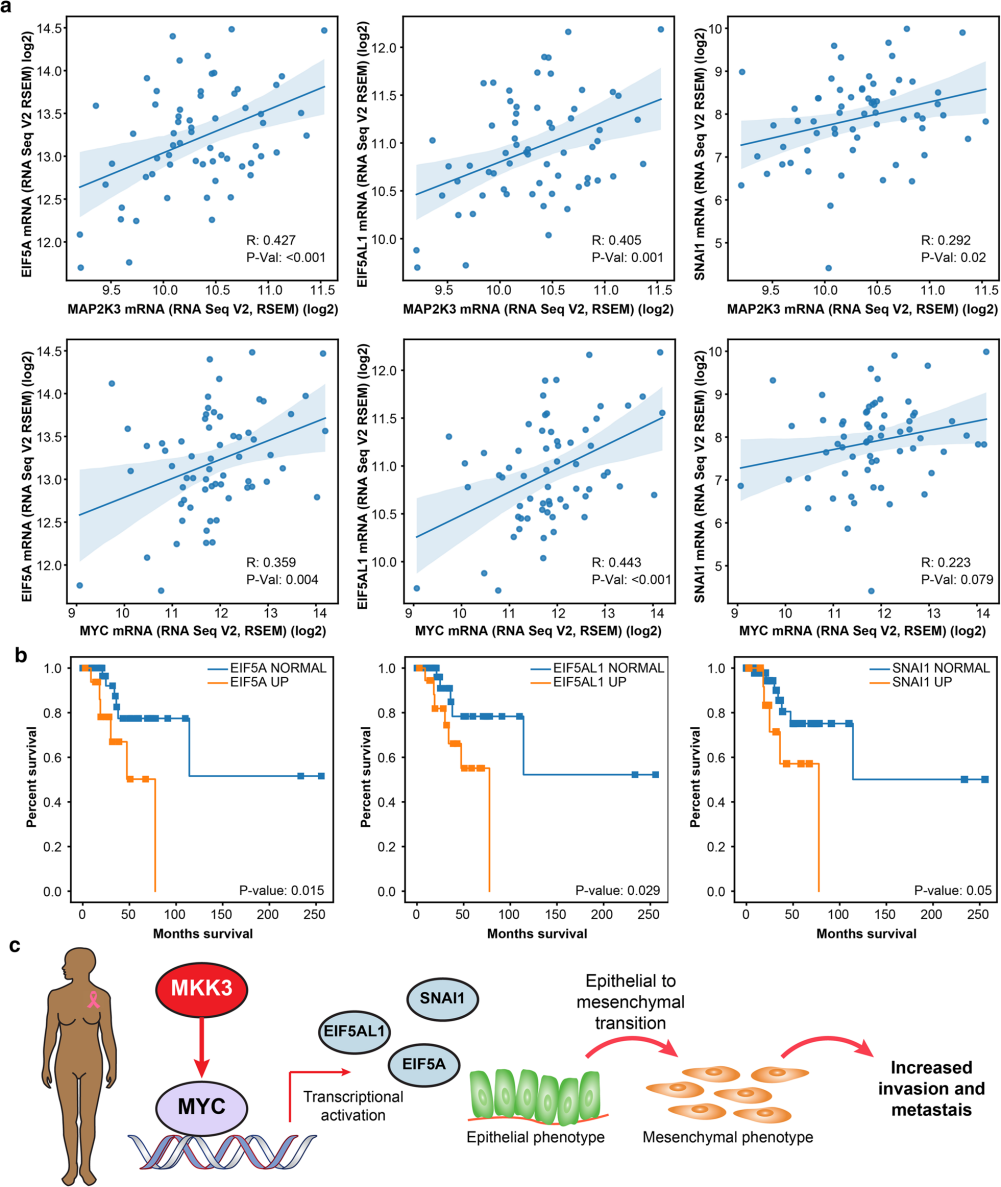
MKK3 overexpression contributes in MYC-dependent induction of epithelial-to-mesenchymal transition. **a** The Pearson correlation plots for the expression of MKK3 (MAP2K3), MYC, and MKK3-MYC interaction and MYC-dependent EMT-inducers EIF5A, EIF5AL1, and SNAI1. **b** Overexpression of EIF5A, EIF5al1, and SNAI1 correlates with decreased survival of African American TNBC patients. Log-rank p-values are shown. **c** A working model by which MKK3-mediated MYC activation leads to transcriptional activation of EMT inducers resulting in increased invasion and metastasis in African American TNBC patients

To identify which of MKK3-activated MYC-regulated genes can contribute most in poor clinical outcomes of African American patients, we evaluated the correlations between overexpression of MKK3-MYC core enrichment genes and TNBC patient survival. We prioritized 8 MKK3-MYC core enrichment genes whose overexpression correlates with decreased survival of TNBC African American patients, including EIF5AL1 (log-rank test p-value = 0.029), EIF5A (p-value = 0.015), SNAI1 (p-value = 0.050), TAF12 (p-value = 0.004) as well APEX1 (p-value = 0.001), FASN (p-value = 0.033), HNRNPA2B1 (p-value = 0.036), and GRSF1 (p-value < 0.001). Notably, overexpression of these genes does not worsen clinical outcomes in Caucasian TNBC patients (p-values > 0.1), suggesting their unique functions in African American patients. We found that EIF5A, EIF5AL1, and SNAI1 are the most frequently overexpressed genes (> 20%) in African American TNBC patients. These genes also demonstrate the highest correlation with both MKK3 and MYC expression (Pearson correlation p < 0.01, Fig. 7a) and decreased survival of African American patients (Fig. 7b). Importantly, both Snail Family Transcriptional Repressor 1 (SNAI1) and Eukaryotic Translation Initiation Factor 5A (EIF5A) have been associated with the induction of the epithelial-to-mesenchymal transition (EMT) in breast cancer, promotion of breast cancer metastasis, and chemoresistance [66–68]. Together, these findings suggest a new function for MKK3 as an inducer of MYC-dependent epithelial-to-mesenchymal transition in African American TNBC patients (Fig. 7c).

## Discussion

Breast invasive carcinoma is the most common cancer type in women. It is especially aggressive in African American patients. The discovery of new therapeutic targets is urgently needed to decrease breast cancer mortality and reduce the racial disparity in breast cancer outcomes. In contrast to the tumor suppressor genes, such as TP53 or BRCA1/2, that are lost due to deletions or mutations, the mRNA overexpression represents an actionable alteration that can be reached therapeutically. The identification of therapeutically actionable upregulated genes that contribute in poor clinical outcomes may facilitate the development of new clinical strategies in breast cancer. Toward this goal, we have performed a systematic analysis of clinical outcomes and differential gene expression in White and African American breast cancer patients.

We found that more than 2500 genes overexpressed in African American patients are also significantly upregulated in African Americans as compared to the White breast cancer patients. Our analysis has also confirmed 117 out of 142 (82%) genes previously reported as differentially expressed in African American and White/European cohorts of breast cancer patients [12]. The enrichment analysis revealed a strong functional association of these genes with breast cancer as well as several other cancer types, and multiple key oncogenic pathways including cell cycle, PI3K-AKT, and JAK-STAT pathways. Through the gene co-expression analysis integrated with the analysis of pathway overrepresentation, we determined specific modules of co-regulated genes. Notably, three distinct gene modules of genes with higher expression in African American patients as compared to White patients were significantly enriched in genes that control cell cycle progression, immunodeficiency, and oxidative phosphorylation. The identification and targeting of the key druggable regulators of these fundamental oncogenic processes may facilitate the development of new clinical strategies to reduce survival disparity in breast cancer. Meanwhile, the diversity of differentially expressed genes and dysregulated pathways (summarized in Additional file 1: Table S3) indicate the heterogeneity and complexity of the molecular mechanisms underlying survival disparity in breast cancer. Thus, the discovery and prioritization of the most biologically clinically essential genes is critical to facilitate the translation of breast cancer patient genomics data into the clinic.

Through the rigorous statistical analysis, we prioritized 32 proteins that demonstrate the most prominent and race-specific association with decreased survival of African American women. We found that 14 of these prioritized proteins belong to proteins classes known to be druggable and thus represent promising targets for therapeutic discovery. Indeed, at least two proteins, ITPA and GPT2, are well-established therapeutic targets for rheumatoid arthritis and anxiety disorders with the FDA-approved inhibitors, azathioprine and phenelzine, respectively. Currently, phenelzine is also in clinical trials in patients with different cancer types, including patients with advanced or metastatic breast cancer. Our data may open new opportunities for the repurposing of these approved drugs and other reported inhibitors as the anticancer agents for African American breast cancer patients.

Through the COX regression analysis, we have identified several proteins as new promising targets for the therapeutic discovery in TNBC. The multivariate COX regression model suggests that expression of MKK3, AGAP2, and ACYP2 has a significant negative impact on clinical outcomes of TNBC Black/African American patients, and these genes may serve as putative biomarkers for decreased patient survival. Among them, MKK3 showed the most dramatic impact on the survival of African American patients, specifically with triple-negative breast cancer.

The integration of the survival data analysis, gene set enrichment analysis, and the analysis of the breast cancer histopathological images revealed that MKK3 can promote TNBC tumorigenesis through the activation of the MYC transcriptional program.

MKK3 is a well-established activator of the p38 pro-inflammatory and pro-apoptotic pathway [58], and MKK3 functions have been associated primarily with the regulation of p38 signaling [69–74]. The loss of p38-activation may promote tumor growth in cancers with a decreased level of MKK3 [57, 75], suggesting its tumor-suppressive function. On the other hand, the oncogenic role for MKK3 has been reported in multiple tumor types, including melanoma, colorectal, liver, esophageal, cervical, and breast cancers [51, 52, 76–80].

Analysis of genomics data shows that MKK3 is either up- or downregulated in different cancer types and different groups of cancer patients. Thus, MKK3 can play a dual role in cancer: one as a lost tumor suppressor acting through the p38-pathway [81, 82], and another as an oncogene through upregulation of different oncogenic programs, such as the MYC transcription. MYC is a major tumor driver, and the master regulator of multiple key cellular processes, including cell growth and proliferation, immune response, and metabolism. Over the past decades, MYC became a well-established and highly-appealing therapeutic target in breast cancer [83]. Therapeutic regulation of MYC activation may provide new clinical strategies to suppress different oncogenic mechanisms in African American breast cancer patients [84–86].

Recent studies have established strong functional connectivity between TP53 mutations in breast cancer patients and MYC activation [87]. The frequency of TP53 mutations is more than 40% higher in African American patients than in White patients [11, 12]. Furthermore, MKK3 overexpression was linked to TP53 mutations in colon and breast cancer cells [56]. These data suggest that MKK3 may cooperate with TP53 to activate MYC and promote TNBC progression in the Black/African American cohort. This model is further supported by a synthetic lethal relationship [88] and a physical protein–protein interaction observed between MKK3 and MYC in cancer cells [60, 61, 89, 90]. Thus, the MKK3-MYC oncogenic axis may represent a new promising target for therapeutic discovery for African American TNBC patients.

Through a systematic gene set enrichment analysis and clinical outcome profiling, we have discovered a new oncogenic function for MKK3 in African American TNBC patients as an activator of MYC-dependent epithelial-to-mesenchymal transition, specifically through EIF5A, EIF5AL1, and SNAI1 genes. Overexpression of these MKK3-MYC signature genes has been linked with the induction of epithelial-to-mesenchymal transition in breast cancer and strongly correlates with worsened clinical outcomes in African American patients. These findings suggest that the inhibition of MKK3-MYC interaction itself and its downstream-activated genes EIF5A, EIF5AL1, and SNAI1 may provide new therapeutic options for African American patients with triple-negative breast cancer.

## Conclusions

In this study, the relationship between gene expression and survival disparity in breast cancer patients has been investigated. Through the integrative statistical analyses of clinical and genomics data, we identified 32 genes as putative targets for therapeutic intervention in Black or African American breast cancer patients. The success of the translation of these findings into the clinic would certainly rely on the further rigorous experimental validation and can be complicated by diverse molecular mechanisms underlying survival disparity. To facilitate this process, the identification and prioritization of the most biologically relevant, clinically significant, and druggable targets is crucial. Toward this goal, we performed a systematic analysis of the genomics and clinical data available for MKK3 gene that demonstrated one of the most significant negative impacts on the survival of African Americans with triple-negative breast cancer. Through a comprehensive systems biology approach, we have linked MKK3-mediated worsened clinical outcomes in African American TNBC patients with the activation of MYC transcriptional program. We have determined that besides its well-defined function in the p38-inflammatory pathway, MKK3 can induce MYC-dependent epithelial-to-mesenchymal transition in breast cancer patients in part through upregulation of EIF5A, EIF5AL1, and SNAI1 genes. These findings suggest new oncogenic functions for MKK3 in breast cancer and define MKK3-MYC interaction as a promising target to reduce survival disparity in African American TNBC patients.

## Supplementary information

**Additional file 1: Table S1.** Differential gene expression analysis. **Table S2.** Modules of co-expressed genes identified with the Weighted Gene Co-expression Network Analysis (WGCNA). **Table S3.** Gene module pathway overrepresentation analysis. **Table S4.** Breast cancer patient survival analysis for genes upregulated in African American patients as compared to White patients. **Table S5**. Univariate COX regression survival analysis for TNBC Balck/African American patients. **Table S6**. Multivariate COX regression survival analysis for TNBC Balck/African American patients. **Table S7.** Analysis of genomic alterations of MKK3. **Table S8.** Genomic status of MKK3 (MAP2K3 gene) and p38 (MAPK14 gene) in BRCA patients and associated clinical data. **Table S9.** Correlation between MKK3 overexpression and breast cancer patient survival. **Table S10.** MKK3 gene set enrichment analysis using the MSigDB cancer Hallmark sets. **Table S11.** MKK3 gene set enrichment analysis for MYC-regulated gene sets. **Table S12.** Pathway overrepresentation analysis for MYC-dependent genes enriched in TNBC patients with overexpressed MKK3.

**Additional file 2.** Additional figures.

## Data Availability

Training images and annotations are publicly available from TCGA sources.

## Abbreviations

ACR: American College of Radiology
ACYP2: Acylphosphatase 2
ADAT2: Adenosine deaminase TRNA specific 2
AGAP2: ArfGAP with GTPase domain, Ankyrin repeat and PH domain 2
BAA: Black or African American
CHCHD1: Coiled-coil-helix-coiled-coil-helix domain containing 1
CDH1: Cadherin 1
CCNE1: Cyclin E1
EIF5A: Eukaryotic translation initiation factor 5A
EIF5AL1: Eukaryotic translation initiation factor 5A like 1
EMT: Epithelial-to-mesenchymal transition
FDA: The United States Food and Drug Administration
FBXW7: F-Box and WD repeat domain containing 7
GDC: Genomics Data Commons
GSEA: Gene set enrichment analysis
HGNC: HUGO Gene Nomenclature Committee
HSI: Hue-Saturation-Intensity
HUGO: Human Genome Organization
MKK3: Mitogen-activated protein kinase kinase 3
MAP2K3: Mitogen-activated protein kinase kinase 3
MRPL18: Mitochondrial ribosomal protein L18
MST: Mean survival time
MYC: MYC proto-oncogene, basic helix-loop-helix transcription factor
NCI: National Cancer Institute
PIK3CA: Phosphatidylinositol-4,5-bisphosphate 3-kinase catalytic subunit alpha
RPP21: Ribonuclease P/MRP subunit P21
RB1: Retinoblastoma protein 1
SNAI1: Snail family transcriptional repressor 1
TCGA: The Cancer Genome Atlas
TNBC: Triple-negative breast cancer
TP53: Tumor protein 53
WGCNA: Weighted gene co-expression network analysis
WSI: Whole-slide images
ZNRD1: RNA Polymerase I Subunit H, also known as POLR1H
