# Supplementary material for: High expression of MKK3 is associated with worse clinical outcomes in African American breast cancer patients
Source: J Transl Med. 2020 Sep 1;18:334. doi: 10.1186/s12967-020-02502-w (PMC7465409; doi:10.1186/s12967-020-02502-w)
Supplement: Supplementary file 2 — Additional file 2. Additional figures. [file 12967_2020_2502_MOESM2_ESM.pdf]

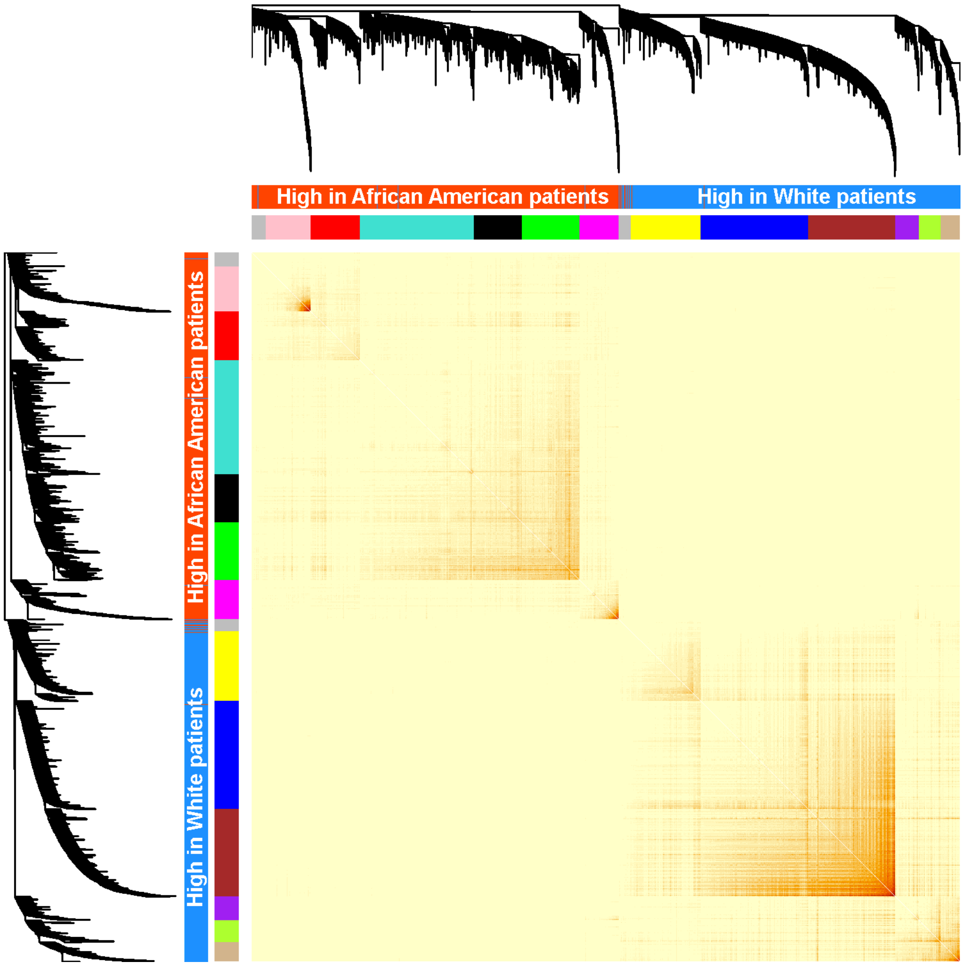

Figure S1. Weighted Gene Co-expression Network Analysis (WGCNA). The heatmap shows the Topological Overlap Matrix (TOM) built for the genes differentially expressed in Black/African American and White breast cancer patients. The darker red color represents the higher overlap. The gene cluster dendrogram is shown on the top and the left side. The middle panel indicates whether the gene expression is higher in Balck/African American (orange) or White (light blue) patients. The colors of the bottom heatmap annotation panel indicate the distinct modules of co-regulated genes.

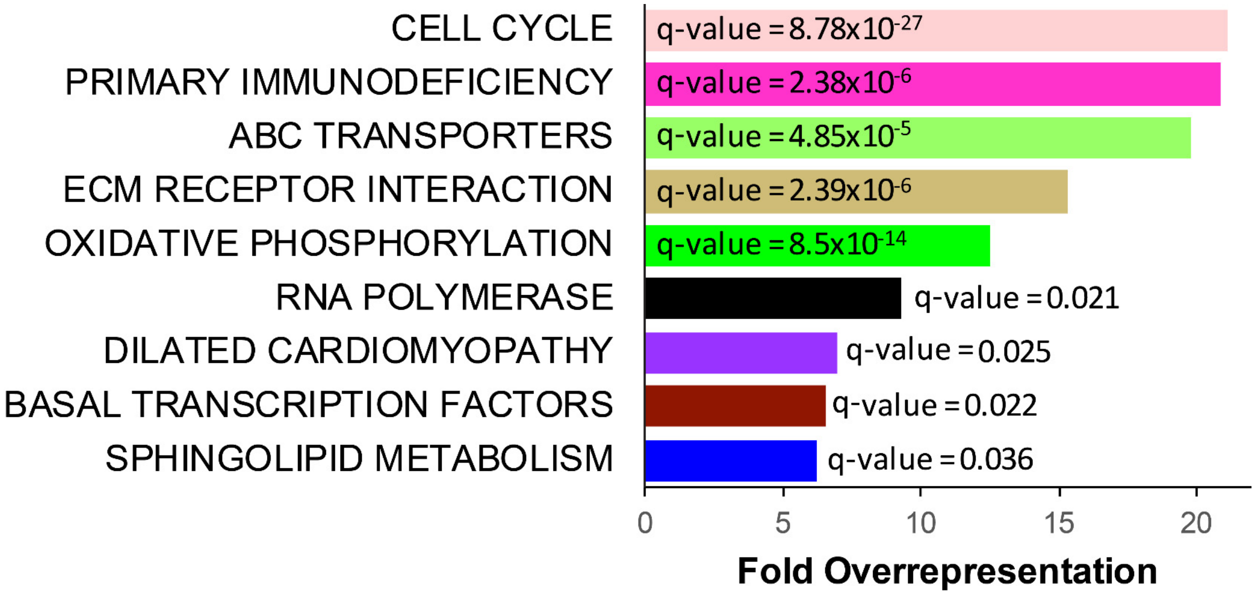

Figure S2. Pathway enrichment analysis performed for the modules of co-regulated genes. The bar color corresponds to the module colors on Fig. S1.

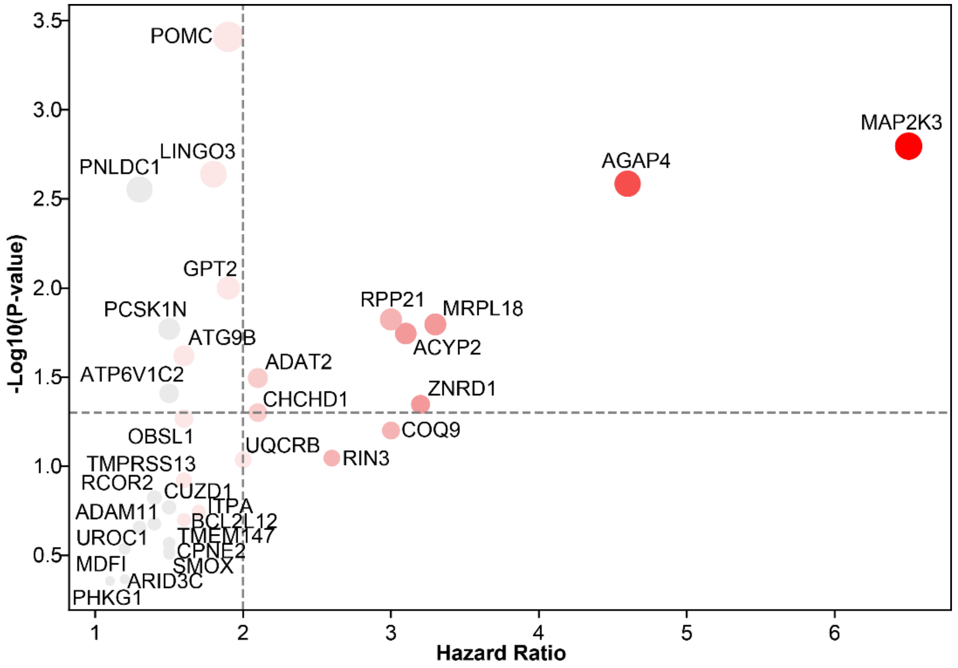

Figure S3. The univariate COX regression survival analysis. The plot shows the relationship between the hazard ratio values and the p-values determined for the prioritized genes in Black/African American TNBC patients. The size of the circles reflects the hazard ratio values, and the color indicate the range of p-values form the less (grey) to the most significant (red). The dash-lines indicate the statistical thresholds for the p-value ≤ 0.05 and the hazard ratio value ≥ 2.
